# Heat Shock Proteins and Autophagy Pathways in Neuroprotection: From Molecular Bases to Pharmacological Interventions

**DOI:** 10.3390/ijms19010325

**Published:** 2018-01-22

**Authors:** Botond Penke, Ferenc Bogár, Tim Crul, Miklós Sántha, Melinda E. Tóth, László Vígh

**Affiliations:** 1 Department of Medical Chemistry, University of Szeged, H-6720 Szeged, Dóm Square 8, Hungary; bogar@sol.cc.u-szeged.hu; 2 MTA-SZTE Biomimetic Systems Research Group, University of Szeged, H-6720 Szeged, Dóm Square 8, Hungary; 3 Institute of Biochemistry, Biological Research Centre, Hungarian Academy of Sciences, H-6726 Szeged, Temesvári krt. 62, Hungary; crul.tim@brc.mta.hu (T.C.); santha.miklos@brc.mta.hu (M.S.); toth.erzsebetmelinda@brc.mta.hu (M.E.T.); vigh.laszlo@brc.mta.hu (L.V.)

**Keywords:** neurodegenerative diseases, neuroprotection, endoplasmic reticulum associated degradation, ubiquitin-proteasome system, autophagy, heat shock proteins, Hsp-inducers, autophagy modulating drugs

## Abstract

Neurodegenerative diseases (NDDs) such as Alzheimer’s disease, Parkinson’s disease and Huntington’s disease (HD), amyotrophic lateral sclerosis, and prion diseases are all characterized by the accumulation of protein aggregates (amyloids) into inclusions and/or plaques. The ubiquitous presence of amyloids in NDDs suggests the involvement of disturbed protein homeostasis (proteostasis) in the underlying pathomechanisms. This review summarizes specific mechanisms that maintain proteostasis, including molecular chaperons, the ubiquitin-proteasome system (UPS), endoplasmic reticulum associated degradation (ERAD), and different autophagic pathways (chaperon mediated-, micro-, and macro-autophagy). The role of heat shock proteins (Hsps) in cellular quality control and degradation of pathogenic proteins is reviewed. Finally, putative therapeutic strategies for efficient removal of cytotoxic proteins from neurons and design of new therapeutic targets against the progression of NDDs are discussed.

## 1. Introduction

Several neurodegenerative diseases, including Alzheimer’s disease (AD), Parkinson’s disease (PD), Huntington’s disease (HD), amyotrophic lateral sclerosis (ALS), and prion disease are, among others, characterized by the presence of specific proteinaceous inclusions in or around the affected neurons. These inclusions are composed of misfolded, aggregated, and often toxic forms of specific proteins (Table 1). Many of these disease-associated proteins are aggregation-prone in nature and easily form misfolded, polymerized structures and toxic aggregates (amyloids) without known physiological functions. Neurons are probably the most vulnerable cells to develop deposits, large inclusion bodies, or aggresomes [1]. The ability of cells to maintain proteostasis—the maintenance of all proteins of the proteome in a conformation, concentration, and location that is needed for their correct function—varies drastically among different cell types [2]. Hence, although the exact mechanism of the particular vulnerability of neurons as post-mitotic cells is not well understood, it is most likely caused by the specificity of the neuronal proteostasis network [3].

Beyond amyloid type protein deposits, neurodegenerative diseases (NDDs) are also characterized by other dramatic pathological changes in the brain. Increased endoplasmic reticulum (ER)-lumen and dysfunction of the endosomal-autophagic-lysosomal pathway, and increased lysosomal membrane permeability occur prior to the development of canonical NDD pathologies [4] and suggest dysfunction of the proteostasis network.

To develop efficient strategies to treat or halt NDDs, it is critical to understand how toxic protein aggregates are cleared from the brain [5,6,7]. Proteostasis pathways maintain the delicate balance between the protection and disposal of proteins. The proteostasis network includes pathways that regulate biogenesis, folding, trafficking, and degradation of proteins (Table 2).

After ribosomal synthesis, nascent polypeptide chains properly fold and assemble into their stable native protein structure. However, approximately 30% of newly synthesized proteins are misfolded and have a high tendency for aggregation [8].

Intracellularly, a complex network of protein quality control processes operates to manage the formation of misfolded proteins. For instance, chaperon proteins such as heat shock proteins (Hsp) assist in the folding, refolding, and recycling of the nascent polypeptide chain in the ER in an ATP-dependent manner [9]. Irreversibly misfolded polypeptides are cleared through ER-associated protein degradation (ERAD) which targets misfolded proteins within the ER for ubiquitination and subsequent degradation by the proteasome (ubiquitin-proteasome system, UPS), and through different autophagy pathways (chaperon-mediated-, macro-, or micro-autophagy). Each pathway degrades substances by lysosomal enzymes [9].

Extracellularly, the formation of toxic protein aggregates is prevented by extracellular enzymatic protein breakdown. In parallel, soluble waste proteins are cleared from the interstitial fluid (ISF) into the blood at the blood-brain barrier through specialized transport systems located in the brain endothelium [10,11] or into the cerebrospinal fluid (CSF) via ISF bulk flow clearance (CSF sink clearance, perivascular drainage, or glymphatic clearance) [12,13].

In this review, we discuss our current progress in understanding the contribution of Hsps, ERAD and UPS, the endo-lysosomal system, and different forms of autophagy in neuronal proteostasis. Especially, the dual role of autophagy in cell survival and cell death will be highlighted. Finally, we focus on the implications of our current knowledge of the neuronal proteostasis processes in drug target development. Considering the role of Hsps in efficient clearance of irreversible misfolded polypeptides, we focus on particular synthetic and natural Hsp-inducers or co-inducers with potential therapeutic value.

## 2. Heat Shock Proteins

### 2.1. Heat Shock Proteins in Proteostasis

As chaperon molecules, Hsps are involved in the maintenance of normal cellular protein homeostasis by regulating the proper folding of newly synthesized peptides and the transport and degradation of mature proteins [14,15]. Members of Hsp-family are involved in each step of proteostasis facilitating protein folding, regulating the rate of protein synthesis and degradation by UPS and autophagy pathways.

However, during exposure to cellular stress (for instance heat shock or oxidative stress) and in certain pathological conditions, there is a fundamental need for an increased chaperon capacity as cells have to cope with enhanced protein misfolding and aggregation. Hence, Hsp levels significantly rise during stress exposure and help to prevent additional conformational changes and self-aggregation of misfolded, partially denatured proteins. In addition, Hsps maintain cell integrity by protecting the plasma membrane [16] while preventing apoptosis by blocking stress kinases [17] or inhibiting the caspase cascade [18].

When the stressful conditions are over, cells try to restore the damages with the help of Hsps which assist in the refolding of misfolded or the degradation of irrecoverable proteins [19].

Formerly, Hsps were grouped according to their molecular weights. However, their increasing number and resulting inconsistencies in their labeling led Kampinga et al. to introduce a new nomenclature of human Hsp families [20]. Currently, Hsps are classified into the following groups: HspH (Hsp110), HspC (Hsp90), HspA (Hsp70), DNAJ (Hsp40), HspB (small Hsps), and the chaperonin families HspD/E (Hsp60/Hsp10) and CCT (TRiC).

The human small Hsp (sHsp/HspB) family has 10 members. They are between 16 and 40 kDa molecular weight and characterized by the conserved C-terminal α-crystallin domain [21]. The activity of HspB proteins is mediated by phosphorylation of serine residues and depends on their oligomeric status. HspB family members are constitutively expressed in several cell types; for instance, crystallins are found mainly in the eye lens as the major structural proteins maintaining the lens transparency [22], whereas HspB5 (αB-crystallin), HspB1 (Hsp27), and HspB2 (MKBP) are highly expressed in cardiac and skeletal muscle cells [23].

As ATP-independent “holdases”, one of their most important functions is binding of misfolded proteins, thereby preventing the formation of insoluble protein aggregates while keeping them available for the “foldase” complexes [24]. HspBs keep their substrates in a near-native state, which can facilitate the refolding [25]. Hence, the observation that the HspBs are found as an integral part of the protein aggregates in vivo led to the novel “aggregase” hypothesis. Accordingly, HspBs can actively sequester proteins during initial unfolding and promote their deposition at specific cellular sites [25].

Although HspB family members are constitutively expressed in several cell types, their expression is upregulated under stress conditions and in diseases, during which they have anti-apoptotic and membrane and cytoskeleton stabilizing properties [26].

Upon recovery after stress, when the ATP level has been restored in the cells, the sequestered damaged proteins are dissociated from the HspB substrate complexes and refolded by the ATP-dependent chaperon machineries [18].

The human HspA/Hsp70 family has 13 members with similar structural and functional properties [20], some of which belong to the most important “foldase” proteins. This family is characterized by constitutively expressed (HspA8/Hsc70), highly stress-inducible (HspA1/Hsp70), and compartment-specific members [27]. For example, the major ER chaperon protein, HspA5/GRP78, has an essential role in the synthesis and folding of newly synthetized proteins followed by their transport to the cytoplasm through the ER membrane. In addition, HspA5/GRP78 is the main regulator of the unfolded protein response (UPR) and is involved in targeting misfolded proteins for ERAD [28].

Members of the HSPA family are ATP-dependent chaperons. In the absence of ATP, HspA family members strongly bind to misfolded protein substrates. Subsequent binding of ATP to the N-terminal region of the chaperon leads to the dissociation of the HspA/substrate complex. This sequential binding and release of the misfolded protein is repeated until its complete refolding [29]. As HspA family members usually have very low basal ATPase activity, the ATP binding and hydrolysis are regulated by several co-chaperons. As an example, HspH/Hsp110 chaperon proteins act as nucleotide exchange factors, removing ADP after ATP hydrolysis [30]. In addition, BAG3 links the ATPase domain of HspA family members to the α-crystallin domain of HspB family members as such increasing their chaperon activity [31].

Members of the human DNAJ/Hsp40 family promote ATP hydrolysis and substrate binding of HspA family members. For instance, DNAJ proteins bind substrate peptides and transfer them to HspA family members meanwhile promoting ATP hydrolysis [32]. In addition, CHIP (C-terminus of heat-shock cognate 70 stress protein-interacting protein) binds to HspA family members as such reducing their ATPase and chaperon activity (Ballinger et al., 1999) [33]. Meanwhile, the E3 ubiquitin ligase activity of CHIP results in the ubiquitination and proteasomal degradation of the client protein previously bound to HspA and HspB family members. In this way, CHIP connects the molecular chaperon and degradation machinery and promotes the degradation of irreversible damaged proteins [34]. The decision of whether misfolded proteins should be refolded or degraded, the so called “molecular triage”, is a central event in the protein quality control and its dysregulation can result in the accumulation of misfolded protein aggregates [35]. For example, CHIP-deficient mice exhibit a markedly reduced life span and accelerated aging. The decreased proteasomal activity and the dysfunction of the protein quality control system in these animals lead to increased levels of toxic protein oligomers in the brain of these animals prior to the emergence of most of the age-related phenotypes [36].

The HspC/Hsp90 family members are the most abundant proteins in cells, representing 1–2% of total cellular protein content [37]. This family of ATP-dependent chaperons has heat inducible and constitutively expressed members in the cytosol, while HspC4/GRP44 is an ER-specific protein. In addition, they are essential for the activation and stabilization of several substrate proteins involved in cellular signaling events, for example by chaperoning steroid hormone receptors protein kinases or the p53 protein [38].

A subpopulation of Hsps is membrane-associated and has a crucial role in membrane quality control while protecting membranes under various stress conditions [16,24,39,40,41]. For example, the mammalian HspB2 was found to be associated with the outer membrane of the mitochondria, whereas a mild heat treatment raised the amount of HspB2 in the mitochondrial fraction [42]. We demonstrated that the 16.2 kDa human Hsp (previously referred as HspB11) associates to lipid membranes through cholesterol controlled interactions, as the efficacy of membrane binding increases in parallel with cholesterol concentration [43]. Moreover, overexpression of this nonconventional small Hsp was found to inhibit cell death through the stabilization of mitochondrial membrane systems [44]. Similarly, association of Hsp17 (a *Synechocystis* protein) with membranes results in an elevated degree of physical order and reduced fluidity [45]. These results suggest that the membrane association of small Hsps contributes to an increased resistance to stress treatments.

### 2.2. Heat Shock Factors Activate the Heat Shock Response

When proteostasis is disturbed, cells try to enhance the cellular chaperon capacity through a rapid heat shock response-mediated increase of Hsp expression levels. This stress-inducible production of molecular chaperons is regulated by heat shock factors (HSF), a family of transcription factors with four members in vertebrates [46]. Next to stress-inducible production of molecular chaperons, HSFs are also important regulators of cell growth and differentiation [47]. Of all HSF members, HSF1 is the most intensively studied and serves as the primary transcription factor to regulate the stress response in almost all cell types.

In unstressed mammalian cells, inactive monomeric HSF1 associates with different Hsps in the cytoplasm. According to the classical model, heat stress-induced formation of misfolded proteins competes with HSF1 for Hsp association. Ultimately, HSF1 is released and quickly undergoes multiple post-translational modifications, trimerizes, and enriches in the nucleus where, upon binding to the heat shock element within the promoter region of stress-inducible genes, it ultimately drives Hsp expression. This entire HSF1 activation cycle is a very rapid process as DNA-binding competent HSF1 can be detected within minutes following heat treatment [14].

The activation and attenuation cycle of HSF1 is strictly regulated by multiple post-translational modifications. However, the heat shock induced DNA-binding of HSF1 is diminished in the brain of old rats compared to young ones what suggests an age-related decline of these regulatory mechanisms [48]. In fact, sirtuin 1 levels—this enzyme deacetylates HSF1 and increases its DNA binding—is reduced in the cortex of AD patients [49]. Next to an impairment of HSF1 regulatory mechanisms, the level HSF1 itself is decreased in the cerebellum of AD model rats [50]. Interestingly, boosting protein quality control through HSF1 overexpression in *Caenorhabditis elegans* delays the onset of polyglutamine (polyQ) protein aggregation while extending lifespan [51].

Additionally, a decreased activity of the protein degradation systems is generally observed in aging what results in the accumulation of aggregation-prone proteins.

Hence, an age-related impairment of the stress response might be involved in the development of certain diseases that are more prevalent in elderly [52].

As the neuronal stress response is considerably weaker compared to other cell types such as glial cells, this could explain the increased vulnerability of neurons to protein misfolding disorders [53,54]. Thus, an age-related overall decline in protein quality control favors the occurrence of neurodegenerative protein-misfolding disorders (Table 1) [55].

### 2.3. Hsp Activation by Membranes as Stress-Sensors

**Figure 1:**
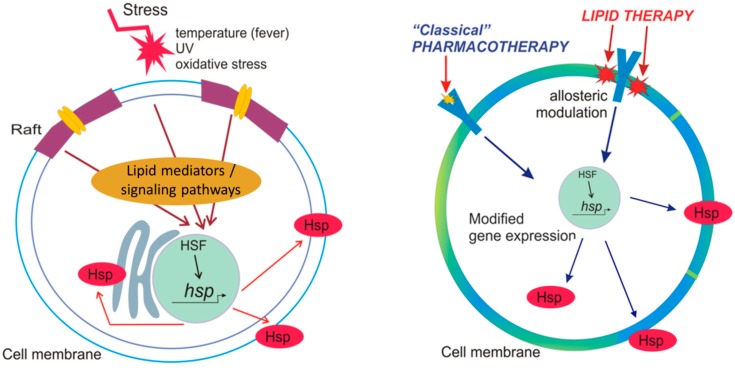
Multiple roles of membranes in stress management: acting as cellular stress sensors, which can also interact with specific Hsps, and as such can serve as a novel target of various pharmacological interventions affecting both the expression and cellular localization/distribution of Hsps.

Stress-induced Hsp-induction in the absence of protein denaturation has been shown in several studies. Hence, alternative thermosensors able to initiate Hsp upregulation should exist. According to the “membrane sensor” hypothesis, the physical properties and microdomain organization of the plasma membrane has a crucial role in the activation of heat shock response [41,56,57]. Since stress factors can influence membrane fluidity and denature membrane proteins, the plasma membrane is a sensitive target for damage under stress conditions and in pathological states. On the other hand, hyperfluidization of the plasma membrane leads to the reorganization of the cholesterol-rich microdomains, which in turn activates the heat shock response [58]. Therefore, increasing the fluidity of membranes (either by heat shock or membrane fluidizers such as benzyl alcohol) leads to the activation of different Hsps. Figure 1 shows the multiple roles of membranes in cell stress, including different mediators and signaling pathways.

Aging and several disorders such as neurodegenerative diseases, diabetes, or cancer are associated with an altered plasma membrane lipid composition and physical properties, such as membrane fluidity. Changes in the membrane composition lead to alterations in the membrane lipid structure and microdomain organization influencing signaling cascades. Consequently, the non-optimal Hsp expression contributes to the development and acceleration of the symptoms of the age-related disorders [59]. Moreover, changes in lipid composition and fluidity of membranes in aging and AD brain affect amyloid binding and make the neuronal membrane more susceptible to β-amyloid (Aβ)-induced injury [60]. The observation that Aβ interacts with membrane lipids, proteoglycans, and several membrane proteins [61] suggests that the Aβ-induced neurotoxic cascade is probably initiated in the cell membrane. Aβ perturbs membrane structure and function altering membrane fluidity; however, there is no agreement on the direction of this effect [62]. Aβ can increase or decrease the membrane fluidity depending on its oligomeric status and on the membrane composition as well. According to the results of Kremer et al. [63], Aβ-induced decrease of membrane fluidity correlates with the aggregation state and surface hydrophobicity. Moreover, the membrane association of Aβ accelerates its own production: Aβ oligomers reduce the membrane fluidity, which in turn stimulates the amyloidogenic processing of amyloid precursor protein (APP), thus generating a vicious circle [64].

According to the “membrane lipid therapy” concept, targeting of certain components of the plasma membrane, such as specific lipids or proteins, can influence signaling pathways by regulating the membrane composition and structure [65]. Hence, membrane intercalating compounds able to pharmacologically modulate the lipid composition or physical properties of cellular membranes represent a promising therapeutic strategy in these diseases. A new class of “membrane lipid therapy” pharmaceuticals exert their beneficial effect by normalizing Hsp expression [59], as discussed in Section 5.

### 2.4. Cytoprotection by Hsps: Prevention of Apoptotic Cascade

Apoptosis or programmed cell death is characterized by the activation of the caspase cascade in which “initiator” caspases induce a chain reaction of specific caspases which cleave and thereby activate each other in a strictly regulated manner. Ultimately, the activated “executioner” caspases degrade cellular proteins which are essential for cell survival. Two major pathways can activate the initiator caspases. The extrinsic pathway is initiated at the cell surface by death receptors, while the intrinsic pathway is induced by pro-apoptotic factors, such as cytochrome c released from the mitochondria [66]. Cytochrome c, by binding to Apaf-1 and procaspase-9, forms the so-called apoptosome, leading to the activation of caspase-9, which in turn activates caspase-3 and initiates the apoptotic protease cascade [67].

Dysregulation of apoptotic cell death is involved in the pathology of different diseases, including NDDs [66]. For instance, the presence of Aβ and α-synuclein induces apoptosis in cultured neuronal cells and in transgenic mice [68]. In addition, increased DNA fragmentation—a characteristic hallmark of apoptotic cell death—was detected in the brain of AD and PD patients, while altered expression of apoptosis-related genes in neurons associates with amyloid plaques [69,70]. In addition, the striatal neurons of HD patients, the spinal cord samples from ALS patients, and the transgenic mouse models of ALS and HD are characterized by caspase activation and increased cytochrome *c* [71,72].

Hsps promote cell survival through protection against changes in the cellular redox homeostasis and stabilization of the cytoskeleton [73]. In addition, Hsps can directly inhibit several steps of the apoptotic pathway [74,75]. In fact, Hsps inhibit the release of pro-apoptotic molecules from the mitochondria while arresting caspase activation [66].

The activity of the stress kinases AKT (protein kinase B) and JNK (c-Jun N-terminal kinase), both modulators of the intrinsic pathway upstream of the mitochondria, is negatively regulated by HspB1 [66]. In addition, direct protein-protein interaction between HspB1 and caspase-3 blocks the cleavage and activation of caspase-3 [76]. HspA1 inhibits apoptosis by binding to Bax as such inhibiting its translocation to the mitochondria [77]. Similar to HspB1, HspC also inactivates AKT kinase [78] while, by forming a cytosolic complex with Apaf-1, it prevents the activation of the apoptosome [79].

This suggests that under pathological conditions, Hsps are potentially important suppressors of apoptotic signaling pathways (Section 5.1).

## 3. ER-Stress, UPR, ERAD, Ubiquitination, and the Ubiquitin-Proteasome System (UPS)

The ER serves many general functions in the cell, including the folding of protein molecules in the cisternae and the transport of synthesized proteins in vesicles to the Golgi apparatus. A large group of soluble and membrane proteins is continuously delivered to the ER as linear polypeptides. The ER lumen thus has a large and varying concentration of nascent unfolded proteins that continuously require processing and folding [80]. Correct folding requires several ER-chaperon proteins (see Section 2), including protein disulfide isomerases, ERdj1, ERdj3, ERdj5, the HspA family member BiP/Grp78, calnexin, calreticulin, as well as members of the peptidylprolyl isomerase family (e.g., cyclophilin B) [81,82]. Only properly folded proteins are transported from the ER to the Golgi apparatus. Several types of disturbances in the cell or cellular environment (calcium and redox regulation problems, glucose deprivation, disturbances in cell membrane, lipid overload [83], over-expression of proteins) can lead to ER-stress responses. In this state, the folding of proteins slows down leading to an increase in unfolded protein level in ER lumen. In ER-stress, the burden of unfolded proteins exceeds the capacity of the ER machinery to deal with them, which ultimately causes widespread protein aggregation and prion formation [84].

Two different fates can thus occur with an unfolded protein in ER: folding or degradation. To restore normal ER-function in protein folding, ER-stress induces two mechanisms: UPR and ERAD.

### 3.1. Unfolded Protein Response

**Figure 2:**
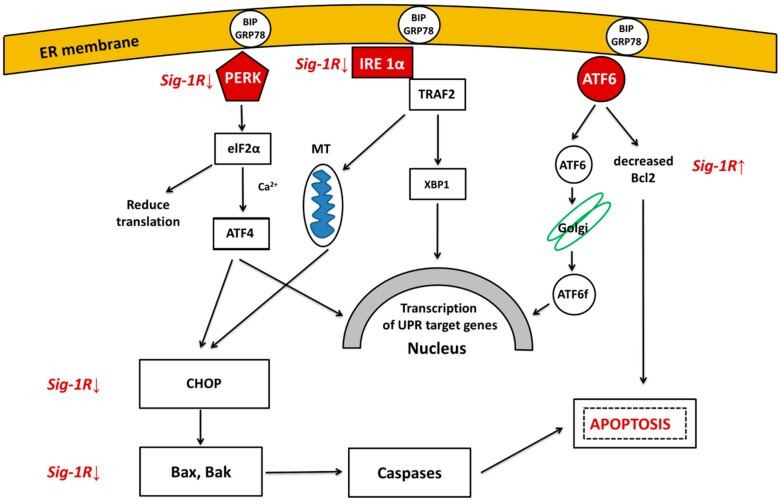
Three signal pathways of ER-stress activate UPR and lead to either cell survival or apoptosis. Under normal conditions, ER chaperon GRP78 binds all the three ER-stress sensors (PERK: protein kinase RNA like ER-kinase; IRE1α: inositol requiring enzyme 1α; ATF6: activating transcription factor 6). Under ER-stress, GRP78 dissociates from the sensors. PERK and IRE1α become phosphorylated and form oligomers, and ATF6 translocates to the Golgi. ATF6, ATF4, and XBP1 activate UPR target genes to enhance the capacity of the ER to cope with unfolded proteins. Activation of Sig-1R inhibits the three branches of UPR. (Abbreviations: eIF2α: eukaryotic translation initiation factor 2α; XBP1: X-box binding protein 1 (spliced form); TRAF2: TNF-associated factor-2; ATF4: transcriptional activator factor-4; MT: mitochondrion).

UPR restores normal function of the cell by halting protein translation, degrading misfolded proteins, and activating different signaling pathways that lead to increased production of molecular chaperons involved in protein folding [85]. UPR is an ER-scanning system by working as a finely-tuned signaling pathway which continuously measures and responds to the ever-changing luminal levels of unfolded proteins [80]. UPR has a cell protective effect as it prevents overload of the ER lumen with newly synthesized proteins and activates degradation of misfolded proteins. However, if the stress signal is severe and/or prolonged, misfolded proteins enter the mitochondria and cause dysfunction in energy production [86,87]. As a result, cell death pathways are triggered in the form of apoptotic and pro-inflammatory reactions [88], as will be detailed below. Figure 2 shows the three major pathways that mediate UPR and are driven by protein kinase RNA-like kinase (PERK), inositol requiring enzyme 1 (IRE1a), or activating transcription factor 6 (ATF6), respectively.

Sustained activation of PERK triggers a signaling cascade leading to C/EBP homologous protein (CHOP) upregulation. This process inhibits the expression of anti-apoptotic B-cell lymphoma 2 (Bcl-2) and upregulates the pro-apoptotic BH3-only proteins. These events result in triggering Bak-(Bcl-2 homologous antagonist killer) and Bax (Bcl-2 like protein 4)-dependent apoptosis [89]. In summary, UPR is a bifunctional cellular response towards protein misfolding with both pro- and anti-survival effects. Basal activity of the UPR is beneficial by activating ERAD for clearing misfolded proteins by UPS and autophagy. However, sustained ER stress and chronic UPR could rather trigger a cell death than cell maintenance program [90]. Chronic ER stress response might be linked to NDDs, such as AD, PD, and HD [89,91]. The neuroprotective effects of Sigma 1 receptor (Sig-1R) agonists modulate all the three branches of UPR and thus show anti-apoptotic effect.

### 3.2. Endoplasmic Reticulum Associated Degradation

**Figure 3:**
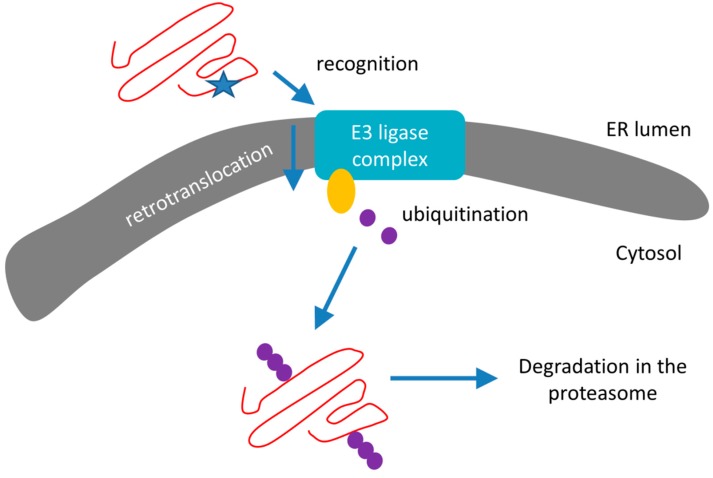
Schematic representation of the ER-associated protein degradation (ERAD) pathway: recognition, retro-translocation, and ubiquitination. Red line: protein/polypeptide chain; blue star: misfolded domain; orange circle: Cdc48 ATPase; purple dots: ubiquitin molecules.

Next to UPR, the ER is also the site of a robust and continuously active protein degradation pathway that recognizes and destroys misfolded forms of both luminal and integral membrane proteins. This so-called ER-associated degradation (ERAD) directs unfolded proteins towards the cytosolic UPS (Section 3.3) and can be divided into three steps (Figure 3):

(1) Recognition of misfolded or mutated proteins in the ER. This process involves detection of substructures within proteins, such as exposed large hydrophobic regions, unpaired cysteine residues, and immature glycans.
(2) Retro-translocation of terminally misfolded proteins into the cytosol. The Hrd1E3 ubiquitin-protein ligase functions as a retrotranslocon (dislocon) to transport substrates to the cytosol. The direction of the transport is determined by the ubiquitin-binding factor Cdc48p in yeast and the valorin-containing protein (VCP/p97) in humans. The energy required for retro-translocation is provided by the ATPase activity of VCP/p97.
(3) Ubiquitin-dependent degradation by the proteasome. Misfolded polypeptides are ubiquitinated by a cascade of enzymatic reactions within the ER membrane, such as the ubiquitin ligases Hrd1 and Doa10 [92]. Next, the polyubiquitinated polypeptide is recognized by specific subunits of the 26S proteasome (and thus ERAD is attached to UPS; Section 3.3) and translocates to the central chamber of the proteasome where the proteolytic active sites are located. ERAD has different branches for different misfolded domains [92].

Indeed, ER-proteins with a misfolded domain in the cytoplasm (ERAD-C substrates) are degraded via the Doa10 complex, whereas proteins containing ER-luminal (ERAD-L) or intramembrane (ERAD-M) misfolded domains are degraded via the Hrd1 complex. Very recently, Zhu et al. [93] demonstrated that the ER-protein membralin is also an ERAD component which mediates degradation of ER-luminal and ER-membrane substrates (ERAD-L and -M). Interestingly, downregulation of membralin results in amyloid-pathology, synaptic deficits, and neuronal death. Hence, membralin might play a critical role in AD pathogenesis.

### 3.3. Ubiquitination and UPS

Ubiquitination destines misfolded polypeptides for degradation through either UPS (Figure 3) or autophagy [94]. Although the ratios between the two protein degradation processes depend on physiological state and cell type, UPS is roughly responsible for approximately 80–90% of cellular proteolysis, whereas autophagy processes only manage around 10–20% [95,96,97]. Misfolded polypeptides are marked for degradation through the attachment of the 76 amino acid long ubiquitin protein. Next, polyubiquitination results in the attachment of additional ubiquitin units on any of the seven lysine residues of the original ubiquitin. This process is mediated by the cooperative action of three enzymes. First, the ubiquitin-activating enzyme E1 hydrolyses ATP and forms a thioester linkage with a cysteine residue of ubiquitin. This activated molecule is then passed on to the E2 ubiquitin-conjugating enzyme. Then, E3 ubiquitin-protein ligases bind to the misfolded proteins and the subsequent sequential ubiquitination of the first ubiquitin residue results in a polyubiquitinated misfolded polypeptide. Hence, ubiquitination generates linkage-specific degrons on substrates destined for destruction [94]. Subsequently, ubiquitin-based degrons are recognized by specific-adaptors with ubiquitin-binding domains (UBDs) [98]. The UBD adaptors RAD23 and UBQLVs deliver ubiquitinated substrates to the central chamber of the 26S proteasome for degradation, whereas the UBD adaptors p62 and NBR deliver ubiquitinated substrates to autophagic vacuoles, phagophores, and lysosomes. As a result, the interplay between polyubiquitination—the diverse ways to assemble ubiquitin chains—and the resulting complex “ubiquitin code” provide several means to modulate biological pathways by proteolytic and non-proteolytic processes [94]. Considering its central role in protein quality control, the UPS is a potential target for the management of AD [99].

### 3.4. Linkage between ERAD and UPR

UPR regulates a large battery of genes that are involved in nearly all aspects of ER protein production and delivery [80]. In fact, multiple genes required for ERAD and involved in the destruction of misfolded luminal and membrane proteins by the UPS pathway (for instance, hrd1/der3, hrd3, der1, and ubc7) are all UPR-regulated. Thus, UPR coordinates the pathways that mediate the two fates of misfolded ER-proteins: either folding and export, or retro-translocation (ERAD) and degradation. Detailed examination of the link between UPR and ERAD places the degradation of proteins in the center of normal ER activity. ERAD is a continuous process and its loss results in an increase of unfolded proteins which are then recognized by the ER-scanning system UPR. However, knock out mouse mutants indicate that ERAD and UPR also function independently to some extent [80].

## 4. Endo-Lysosomal System and Autophagy

### 4.1. Endo-Lysosomal and Autophagy Dysfuntion in NDDs

The endo-lysosomal network contains a lot of communicating vesicular compartments with acidic pH ranging from 4.5 to 6.0. Material from outside the cell is taken up by endocytosis and passed on to the lysosomes, spherical vesicles containing more than 60 different hydrolytic enzymes able to break down proteins and many kinds of other biomolecules. As such, this network regulates cellular homeostasis and metabolism through degradation of cargo that is received by endocytosis from the plasma membrane. In addition, the autophagic pathways sequester cytosolic components, organelles, and toxic amyloids present in the cytoplasm, which are then digested by the lysosomes.

In AD, the endo-lysosomal network is involved in the generation of Aβ through enzymatic cleavage of APP in the lysosomes by β- and γ-secretases (the amyloidogenic pathway) [100,101]. In AD patients, the presence of amyloid plaques blocks the ability of lysosomes to travel within the axons and develop to mature lysosomes [102,103]. As such, these immature lysosomes are abnormally enriched in β-secretase since these lysosomes are not able to degrade this APP-cleaving enzyme. This represents a vicious circle since the increased number of immature lysosomes correlates with a higher local accumulation of β-secretase and a resulting higher amount of Aβ. Thus, accumulation of lysosomes even contributes to AD pathology. Next to lysosomes, Aβ is present in additional subcellular organelles of the degradative pathway such as endosomes, late endosomes/multivesicular bodies, autophagic vesicles, and lysosomes [104,105]. In addition to Aβ, other APP fragments (such as C83 and AICD) as well as aberrant post-translational APP modifications and dysregulation of APP processing contribute to the pathogenesis of AD [106,107,108]. Hence, perturbation of APP metabolism and subsequent Aβ formation in the endo-lysosomal network is a central pathomechanism of AD [109].

Next to the lysosomal symptoms, AD is characterized by an endosomal pathology with increased endosomal size compared to healthy aged brains [104]. In fact, Aβ accumulation in the EL system increases lysosomal membrane permeability and causes the release of the lysosomal content (for instance cathepsins) into the cytoplasm ultimately resulting in apoptotic cell death [110]. In fact, one of the earliest events in Aβ-mediated neurotoxicity is just the release of lysosomal proteases. Interestingly, recent data indicate that the molecular chaperon Hsp70.1 acts as a lysosomal stabilizer [111,112]. The symptoms of the endocytic and lysosomal pathology are among the primary events of AD. In fact, AD patients exhibit endo-lysosomal network pathology in brain regions that do not yet show local Aβ or tau-pathology [113]. Of note, these morphological changes were also observed in the brain of patients with Down syndrome [114].

Extracellular amyloid plaques of AD individuals accumulate endo-lysosomes and organelles of the autophagy network as these plaques are surrounded by dystrophic neurites that are enriched in lysosomal-like organelles [115]. A bidirectional relationship between the endo-lysosomal/autophagic network and AD might exist since the activity of certain lysosomal enzymes (e.g., cathepsin D) increases, whereas the activity of other enzymes decreases [116].

Interestingly, lysosomal storage disorders (LSDs) are also characterized by accumulated APP metabolites. Consequently, AD and LSDs show common neuropathological features as lysosomal dysfunction in LSD leads to a kind of neurodegeneration and dementia similar to AD. Hence, endo-lysosomal dysfunction should be accepted as a potential risk factor for AD [116] and might be a converging pathomechanism in NDDs [117,118].

### 4.2. Autophagy

Autophagy or autophagocytosis refers to the mechanism which allows the cell to destroy and degrade unnecessary and/or dysfunctional components in the lysosome [119]. However, autophagy is a double edged sword. In disease, autophagy pathways represent an adaptive response to stress which promotes survival, whereas in other cases, autophagy promotes cell death. The molecular definitions of autophagy and its related processes were recently reviewed [120]. Three main forms of autophagy are commonly described: chaperon-mediated-autophagy (CMA), macro-, and microautophagy.

CMA is a very complex and specific multistep process. In CMA, soluble cytosolic proteins are selected in a chaperon-dependent manner, targeted to lysosomes, and directly translocated across the lysosomal membrane for degradation. Of note, CMA directly shuttles proteins across the lysosomal membrane without formation of additional vesicles. In addition, CMA differs from other types of autophagy as it is extremely selective towards the proteins which cross the lysosomal barrier.

In CMA, chaperon-bound autophagy substrates bind to the protein-translocation factor LAMP2A monomer on the cytosolic side of the lysosome forming an oligomeric LAMP2A translocation complex [121]. Through the LAMP2A complex, CMA substrates are translocated into the lysosomal lumen while they are unfolding and dissociating from the chaperons. During this process, LAMP2A complexes are stabilized by the lysosomal chaperon Hsp90AA1 and the cytosolic pool of glial acidic fibrillary protein (GFAP) [121]. CMA is responsible for the selective removal of damaged and unnecessary proteins. By facilitating the recycling of amino acids of the degraded (misfolded) proteins, CMA plays an important role in the regulation of the cellular metabolism. Of note, CMA is a continuous process which is active in different tissues and organs, such as brain, liver, and kidney.

**Figure 4:**
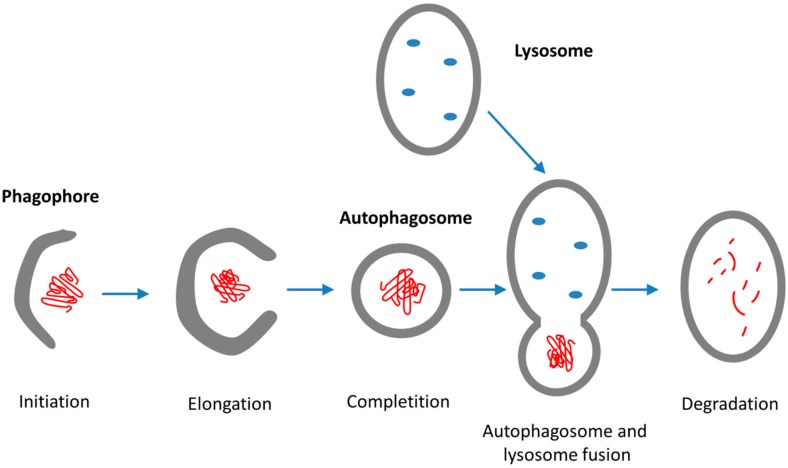
Macroautophagy is a multistep process: initiation, elongation, completion, fusion, and lysosomal degradation. Red coil: misfolded protein; red lines: oligopeptide and amine acids; blue dots: lysosomal enzymes.

Macroautophagy is the main, multistep pathway for degradation of damaged cell organelles and unused proteins with participation of the lysosome (Figure 4). The first step is the formation of a crescent-shaped double membrane (phagophore or isolation membrane) derived from multiple sources, such as cytoplasmic membrane, mitochondria, or ER- and Golgi apparatus [96,122]. During the following steps, the double-membrane phagophore grows in size and closes to form a complete autophagosome. Next, the membrane of the autophagosome fuses with lysosomes to form autolysosomes. Finally, the cargoes (e.g., misfolded polypeptides) are degraded by lysosomal hydrolases.

In microautophagy, cytoplasmic entities destined for degradation are directly taken up by lysosomes via direct membrane invagination, without formation of protein complexes [123]. Although microautophagy is not well studied, two forms of this pathway were recently defined: microautophagy and endosomal microautophagy. The non-selective microautophagy begins with invagination of the cytosolic substrate. It is a tubular process by which the autophagic tube is formed. Endosomal microautophagy relies on multiple endosomal sorting complexes required for transport (ESCRT) systems [124]. In mammalian cells, this mechanism involves mainly late endosomes. Endosomal microautophagy degrades cytosolic proteins. Selectivity of this autophagy requires chaperon interaction, for instance only proteins containing a Lys-Phe-Glu-Arg-Gln (KFERQ-like) motif are recognized by Hsp78 [124].

**Figure 5:**
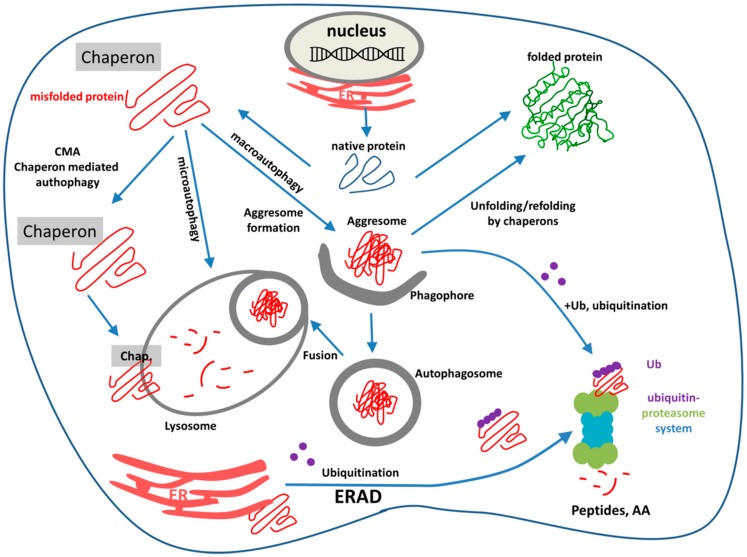
The most important pathways of intracellular protein degradation: endoplasmic reticulum associated-degradation (ERAD), the ubiquitin-proteasome system (UPS), and autophagy pathways. Purple dots: ubiquitin molecules; red coil: misfolded aggregated proteins; red lines: oligopeptides or amino acids.

The three types of autophagy are regulated by similar signaling pathways induced by starvation, nitrogen deprivation, rapamycin treatment, etc. Autophagy is executed by autophagy-related (Atg) genes. Recent studies focusing on the relationship between aging and autophagy gave interesting results: senescence of cells was associated with decreased autophagy and decreased expression of autophagy genes [125,126]. It occurred as the result of the increased methylation of autophagy genes by the DNA methyl transferase DNMT23. The autophagy gene ATG5 was one of the highly methylated autophagy genes. In mammals, Atg1 complex is based on the kinases ULK1 or ULK2, and plays an essential role in regulating autophagy [127]. ULK is part of a larger protein complex containing autophagy-related proteins mAtg13, Atg101, and FIP200. The ULK complex is regulated by a series of phosphorylations on ULK1/2, mAtg13, and FIP200. The ULK1/2 function (and autophagy) is regulated by mTOR complex1 and 5′-AMP-activated protein kinase (AMPK) through a complicated network of phosphorylation events on a large number of phospho-sites of ULKs Interestingly, nutrient starvation prolongs lifespan in *C. elegans* through the induction of caloric restriction in parallel with a high level of autophagy, an effect which can be simulated by resveratrol [128]. Figure 5 schematically represents the cellular fate of misfolded polypeptides indicating ERAD, UPS, and the three different autophagy pathways.

## 5. Prevention and Treatment of NDDs

### 5.1. Heat Shock Proteins in Neurodegenerative Disorders

Many neurodegenerative diseases are characterized by the accumulation of misfolded proteins in different forms: intra- and extracellular aggregates, plaques, inclusion bodies, and proteinaceous fibrillary structures (Table 1).

The formation of extracellular aggregates is mainly a tool for sequestration of toxic, pathogenic proteins since, in case of Aβ, the soluble oligomers and protofibrils are the most cytotoxic forms [129,130]. In fact, according to the intracellular Aβ hypothesis, intracellular Aβ oligomers initiate the disease by interacting with cytoplasmic proteins and membranes of cell organelles (mitochondria, ER), thereby triggering apoptosis [131]. In addition, polyQ proteins interact with transcription factors, proteasomal subunits, and cytoskeletal proteins eventually leading to repression of transcription, impairment of the protein degradation system, and alteration of the neurofilament network [132]. Although Aβ plaques do not appear to be as neurotoxic as the soluble oligomers, they do provoke local inflammatory responses. Ultimately, this leads to uncontrolled activation of microglia and release of inflammatory cytokines and thus chronic inflammation in the brain [133].

Due to a different and neuron-specific protein quality control system compared to glial cells, ubiquitously expressed amyloids are selectively deposited in neurons [134]. Hence, compared to neurons, glial cells are much less affected in neurodegenerative diseases. In fact, neurons have a lower basal level and a weaker heat-inducibility of HspA1 compared to astrocytes, whereas astrocytes have higher CHIP activity and degrade toxic, aggregation-prone proteins faster than neurons [53].

In parallel with the increasing level of misfolded polypeptides, Hsp expression levels are usually upregulated in the affected brain regions. For instance, the levels of HspB1 and HspB5 are highly elevated in the cerebral cortex of AD patients [135,136], in the reactive astrocytes in PD [135], and in the spinal cord in a mouse model of ALS [28]. Thus, the levels of several Hsps are constitutively increased in neurodegenerative diseases as a compensatory mechanism.

In addition, Hsps co-localize with the abnormal protein aggregates (reviewed by [26,137])*.* In fact, several members of the small Hsp family, such as HspB1, HspB2, HspB5, and HspB6 associate with senile plaques in the AD brain [138]. In addition, HspB5 is mainly localized in astrocytes, microglia, and oligodendrocytes, while HspB1 was found in degenerating neurons [136,139]. HspB5 localizes to the Lewy bodies [140] and is present in the spinal cord of ALS patients [141]. In addition, HspA1 co-localizes with Aβ peptides and α-synuclein, whereas HspA8 was found in intracellular inclusions in ALS [142].

In certain cases, aggregation-prone polypeptides evade the protein quality control systems. In fact, due to the increasing rate of aggregate formation, Hsps get trapped within the aggregates what reduces their availability. For instance, an increased level of aggregate formation correlates with a decreased ability of small Hsps to prevent α-synuclein fibril formation in vitro [143].

In parallel, the inducibility and chaperon activity of Hsps are impaired during aging (reviewed in [55]) which is accompanied by a reduced activity of the protein degradation systems as well [144]. Hence, the combined effect of the weakening of the capacity of the chaperons and the protein degradation complex as well as an increased formation of misfolded proteins in aged organisms finally leads to the overload of the system what further accelerates the development of NDDs [55].

Therefore, restoration of the heat shock response and the increase of chaperon activity of Hsps represent useful therapeutic strategies. In fact, each neurodegenerative disorder is characterized by a specific subset of Hsps able to ameliorate the specific symptoms [145]. Consequently, the protective effect of elevated levels of Hsps in different animal models of NDDs has been shown. For instance, HspB1 overexpression delays the decline of motor strength and improves the survival of the spinal motor neurons in a transgenic mouse model of ALS [146]. In addition, we showed that HspB1 overexpression rescues the impaired learning abilities, decreased the number of amyloid plaques, and normalized synaptic abnormalities, such as increased excitability and impaired long-term potentiation, in a mouse model of AD [147]. Overexpression of HspA1 prevents α-synuclein-induced dopaminergic neuronal loss [148] and polyQ-induced neurodegeneration [149] in *Drosophila* and in SCA1 transgenic mice [150]. In addition, transgenic overexpression of HspA1A reduces Aβ plaque formation, neuronal loss, and cognitive deficits in a mouse model of AD [151]. These results indicate that overexpression of Hsps ameliorates certain symptoms of neurodegenerative diseases.

The protective function of Hsps in NDDs lies in their capacity to suppress pathological protein aggregation via their chaperon-like activity. This potent anti-aggregation property of Hsps is well documented by in vitro studies, both in experiments performed with solutions of aggregation-prone proteins and in cell culture experiments [152].

In fact, several studies demonstrate that even individual Hsps can prevent protein aggregation. For instance, HspB1, HspB5, and HspB8 bind α-synuclein and inhibit mature fibril formation [153]. Mechanistically, HspB1 and HspB5 prevent aggregation through transient interactions with α-synuclein monomers as such stabilizing their structure and inhibiting the formation of oligomeric nuclei [143]. Similarly, by binding to fibril seeds rather than forming a stable complex with the monomeric amyloid peptides, HspB5 prevents the induction of β-sheet structure and the amyloid fibril growth of Aβ1-40 [154]. In addition, HspB1 inhibits Aβ1-42 amyloidogenesis in vitro [152]. Most of the in vitro studies show that addition of individual Hsps to the reaction after the completion of aggregation has no effect on the size of aggregates, which suggests that Hsps bind to monomeric and/or prefibrillar forms of aggregation prone proteins as such keeping them in a soluble form which prevents fibril growth.

Several cell culture models provide evidence that Hsps prevent abnormal protein aggregation. For instance, overexpression of DNAJ protein family members in cerebrovascular cells suppresses the nuclear aggregation of mutant ataxin-1 and ataxin-3 [129,155], whereas HspB1, HspB5, and HspB6 are able to bind to Aβ inhibiting its fibril formation [156]. In addition, HspB1 and HspB5 expression can significantly reduce α-synuclein aggregation [157]. Of note, the capacity of small Hsps to reduce α-synuclein inclusion formation is retained even in the presence of inhibitors of autophagy or proteasomal degradation [143]. Hence, by preventing initiation of aggregation, Hsps are potent inhibitors of protein aggregation in living cells. Interestingly, in most of these studies, the reduced aggregation was accompanied by a decreased toxicity of the aggregation-prone polypeptides.

Small Hsps family members have several additional non-chaperon functions that could contribute to their neuroprotective function. In fact, small Hsps are potent inhibitors of the apoptotic cascade. In addition, the increased level of Hsps can prevent apoptotic cell death induced by aggregation-prone proteins, such as α-synuclein or Aβ [158,159]. Hence, since neurological disorders such as AD, PD, HD, and ALS are characterized by enhanced apoptotic cell death, the neuroprotective function of small Hsps might lie in their capacity to prevent apoptosis [160,161,162].

Cellular oxidative stress increases during aging and in different NDDs. For instance, HspB1 has been shown to decrease polyQ toxicity without suppressing protein aggregation by protecting cells against oxidative stress indicating that Hsps can regulate cellular redox homeostasis and have protective effects against oxidative stress independently from their anti-aggregation effect [163]. Similarly, Hsp70.1 has dual roles: it acts as a molecular chaperon for damaged proteins while simultaneously it is a guardian of lysosomal integrity. It is known, that in the acidic environment of the lysosome lumen, Hsp70.1 interacts with bis(monoacylglycero)phosphate (BMP), an anionic phospholipid bound to the inner lysosomal membrane. BMP is a cofactor for the enzyme acid sphingomyelinase (ASM). The Hsp70.1-BMP interaction enhances the association of BMP with ASM, activating the enzyme so that it breaks down sphingomyelin to form ceramide. Thus, the increased production of ceramide in lysosomes protects lysosomal membranes from rupturing [40]. According to the “calpain-cathepsin hypothesis” of AD [111], both age-dependent oxidative stress and decreasing Hsp70.1 levels might be responsible for elevated lysosomal membrane permeability and cell death. Specific membrane lipids containing linoleic and arachidonic acids are vulnerable to cumulative oxidative stresses, especially because their toxic peroxidation product 4-hydroxy-2-nonenal (HNE) preferentially carbonylates Hsp70.1, and, in the next step, calpain cleaves and inactivates this carbonylated Hsp70.1 pool. This event ultimately causes elevated lysosomal permeabilization and rupture, with the release of cathepsins and finally cell death [112].

While essential for the normal neuronal function, the dendritic network is usually altered in NDDs. For instance, in AD patients, dendritic degeneration is remarkable characterized by a significant loss of total dendritic length and the density of dendritic spines [164,165]. However, during neuronal stress conditions, the upregulated HspB5 plays an important role in the preservation of dendritic complexity and neuronal connectivity by stimulating dendritic branching [166].

### 5.2. Targeting Hsps for Treatment of NDDs

Misbalanced Hsp expression levels are central in the pathogenesis of multiple prevalent disorders. Hence, compounds able to restore Hsp expression levels have high therapeutic potential. It has been demonstrated that overexpression of HspA1A and HspB1 proteins in a transgenic mouse models of AD (APPxPS1 mouse) can ameliorate the major symptoms of AD by improving synaptic and cognitive functions, decreasing the number of amyloid plaques, and increasing neuronal survival [147,151]. These results indicate that chaperon molecules are potential targets in AD therapy and subsequently have drawn attention to search for chaperon inducer and co-inducer molecules, both natural plant extracts as well as synthetic small molecules. However, only diseased tissues or cells should be targeted as a general increase in Hsp expression levels correlates with carcinogenesis. As a therapeutic class, Hsp co-inducers cannot amplify Hsp expression without a concomitant stress. However, they further elevate Hsp levels in the presence of cellular stress underlying the phenotype of several pathological conditions. Here, we describe some selected compounds that exert neuroprotective properties via chaperon induction/co-induction. A comprehensive review on the protective role of plant biophenols in mechanisms of AD was recently published [167].

#### 5.2.1. Therapeutic Potential of Small Molecule Hsp Co-Inducers

Hydroximic acid derivatives including bimoclomol, arimoclomol, NG-094, and BGP-15 are nontoxic small molecules based on the structure of the beta-blocker propranolol [59].

Vigh et al. were the first to demonstrate the Hsp co-inducing capacity of hydroximic derivatives by showing that the first generation derivative bimoclomol, a hydroxylamine derivative ((2-hydroxy-3-(1-piperidinyl) propoxy)-3-pyridinecarboximidoil-chloride maleate), synergistically enhances heat-induced Hsp60, Hsp70, and Hsp90 levels [168]. The compound facilitates the formation of chaperon molecules in eukaryotic cells by inducing or amplifying expression of heat-shock genes. The cytoprotective effects observed under several experimental conditions, including a murine model of ischemia and wound healing in the diabetic rat, are likely mediated by the coordinate expression of all major Hsps. Consequently, potential therapeutic value for bimoclomol was established in diabetes [169,170] and cardiovascular disease [171,172,173], pathologies characterized by an imbalance in Hsp expression.

Next, arimoclomol((3*Z*)-*N*-((2R)-2-hydroxy-3-piperidin-1-ylpropoxy)-1-oxidopyridin-1-ium-3-carboximidoyl chloride) reduces lysosomal accumulation in primary fibroblasts from patients with lysosomal storage diseases, a pathology associated with severe systemic and central nervous system symptoms [174]. In addition, in a rhodopsin transgenic rat models, a model for retinitis pigmentosa—a group of inherited diseases that cause blindness due to the progressive death of rod and cone photoreceptors in the retina, pharmacological potentiation of the stress response with arimoclomol improved electroretinogram responses and prolonged photoreceptor survival [175]. In a mouse model of spinal and bulbar muscular atrophy, an adult-onset hereditary neurodegenerative disorder caused by an expansion of polyQ repeats in the first exon in the androgen receptor gene, arimoclomol significantly improved hindlimb muscle force and contractile characteristics, rescued motor units and improved motor neuron survival while upregulating the expression of the vascular endothelial growth factor which possess neurotrophic activity [176]. In a mouse model of ALS, an incurable neurodegenerative disorder characterized by progressive degeneration of motor neurons leading to death, arimoclomol preserved neuromuscular function and endplate size while delaying disease onset and improving lifespan [177,178,179]. Clinical trials showed that arimoclomol is safe and tolerable for ALS patients [180]. A clinical Phase II trial of arimoclomol in SOD1 mutant ALS-patients has been completed recently [181]. The benefits of arimoclomol are not limited to neurons as the drug candidate also restores muscle function and reduces protein aggregation in a mouse model of inclusion body myositis—an inflammatory muscle disease characterized by slowly progressive weakness and wasting of both distal and proximal muscles [177].

In a *C. elegans* model of HD, a neurodegenerative disorder caused by aggregation-prone polyQ expansion proteins, NG-094 ameliorates polyQ-mediated animal paralysis, reduces the number of polyQ aggregates and delayed polyQ-dependent acceleration of aging [182].

Finally, BGP-15 (*O*-(3-piperidino-2-hydroxy-1-propyl)-nicotinic amidoxime) protects against tachycardia remodeling in Drosophila through endogenous overexpression of Dhsp23 [183]. In addition, BGP-15 improves insulin sensitivity in multiple animal models of type 2 diabetes [184,185,186,187,188,189,190,191] and in insulin-resistant, nondiabetic patients [192]. In a mouse model of muscular dystrophy, BGP-15 decreases kyphosis, improved the dystrophic pathophysiology in limb and diaphragm muscles, and extends lifespan [193,194]. BGP-15 was found to accumulate in the mitochondria, protect against ROS-induced mitochondrial depolarization and attenuate ROS-induced mitochondrial ROS production in a cell culture model, as well as reduce ROS production predominantly at the complex I to III system in isolated mitochondria. At physiologically relevant concentrations, BGP-15 protected against hydrogen peroxide-induced cell death by reducing both apoptosis and necrosis. BGP-15 protects the brain against cellular apoptosis, reduces inflammatory cell infiltration and gliosis, while improving behavior in a traumatic brain injury mouse model [195]. BGP-15 is safe and well tolerated in insulin-resistant, nondiabetic patients [192].

At the molecular level, hydroximic acid derivatives interact with lipids. Bimoclomol interact with acidic lipids and acts as a membrane fluidizer at normal temperature, whereas, during severe heat shock, it stabilizes the membrane through inhibition of bilayer-nonbilayer phase transitions [196]. Furthermore, BGP-15 modulates plasma membrane nanodomains (lipid rafts): on the one hand in vitro during heat shock suggesting modulated lipid raft-originating signaling cascades which ultimately act on HSF1 [197,198,199] and in a mouse model of atrial fibrillation on the other [200]. In parallel, bimoclomol and arimoclomol increase HSF1 activity through hyperphosphorylation [179,201] resulting in enhanced HSF1 binding to its DNA recognition element [201]. In addition, BGP-15 influences the activation and attenuation of HSF1 upon stress through inhibition of histone deacetylases, resulting in increased chromatin accessibility and a decrease of total HSF1 acetylation [199,202,203].

This seemingly “random” multi-target profile of hydroximic acid derivatives might in fact underlie their strength. Several diseases are described as a network phenomenon in which the partial inhibition of a small number of targets can be more efficient than the complete inhibition of a single target [204,205]. Hence, multi-target drugs might have a better chance of affecting the complex equilibrium of whole cellular networks than drugs that act on a single target [204]. Thus, it is plausible that the chaperon co-inducing property of the hydroximic acid derivatives consists of combinatorial effects occurring in the plasma membrane and intracellular compartments.

Plasma membrane micro-heterogeneity is important for regulating the Hsp response. In fact, the structure of plasma membrane-residing lipid rafts is strongly dependent on the thermally controlled lipid phase behavior. Hence, even mild changes in temperature could result in a fundamentally altered fluidity and, consequently, in the redistribution and activity of potential stress-sensing and/or stress-signaling proteins within these subdomains [57,58,197,206,207,208,209,210,211,212,213,214,215]. Docosahexaenoic acid shows neuroprotective effects mainly by decreasing de novo cholesterol biosynthesis and shifting cholesterol from the rafts to the non-raft fraction [216]. A variety of diseases are characterized by plasma membrane abnormalities which might exacerbate their pathogenesis or hamper a cellular defense response [65]. Thus, membrane intercalating compounds such as hydroximic acid derivatives have the potential to become a new class of pharmaceuticals for use in “membrane-lipid therapy” which can be used to alter membrane properties and ultimately restore the Hsp profile [65,217].

Next to hydroximic acid derivatives, dihydropyridine derivatives are a new class of Hsp co-inducing compounds. The dihydropyridine derivatives LA1011 and LA1044 enhance the heat-induced expression of Hsp70, Hsp27, and Hsp40 in SH-SY5Y human neuroblastoma cells. In addition, six-month administration of LA1011 improved the spatial learning and memory functions in wild type mice, whereas it eliminated neurodegeneration by increasing dendritic spine density and reducing tau pathology and amyloid plaque formation in APPxPS1 double mutant mice, a mouse model of AD [218].

Steryl glucosides are widespread membrane-bound sterol derivatives in many organisms [219]. Upon stress exposure, cholesteryl glucoside is rapidly formed in animal tissues and human cultured cells [220]. Exogenously addition of cholesteryl glucosyl induces HSF1 activation and Hsp70 production in animal tissue and human fibroblasts [221]. At the molecular level, the cholesteryl glucoside synthase glucosyltransferase resides in lipid rafts [222]. Hence, it is suggested that heat stress-induced melting of the lipid rafts releases the enzyme from tight packing resulting in a conformation change and activation of the enzyme [222]. Consequently, stress-induced glycosylation of cell membrane cholesterol results in a reorganization of sterols as well as other membrane components subsequently having an effect on the cellular signal transduction machinery [222,223].

Hsp90 inhibitors are currently used in clinical trials for multiple cancers [224]. As a side effect, Hsp70 induction is frequently observed which might limit their use [225]. Under normal conditions, monomeric HSF1 is kept in an inactive complex through binding to, among others, Hsp90 [226]. In the presence of Hsp90 inhibitors, this inhibitory complex falls apart, HSF1 trimerizes and enriches in the nucleus where it ultimately drives Hsp70 expression, among others. Through this mechanism however, Hsp90 inhibitors have therapeutic potential in neurodegenerative pathologies. In fact, in spinal and bulbar muscular atrophy, polyQ-expanded mutant androgen receptor—an Hsp90 client protein—adopts a misfolded conformation that tends to aggregate in neurons. Administration of the Hsp90 inhibitors 17-AAG (17-allylamino-17-demethoxygeldanamycin) or 17-DMAG (17-(dimethylaminoethylamino)-17-demethoxygeldanamycin) markedly ameliorates motor impairments in a mouse model of spinal and bulbar muscular atrophy while reducing mutant androgen receptor levels. In parallel, 17-AAG and 17-DMAG mildly upregulated Hsp70 and Hsp40 expression levels, the former of which might represent an additional therapeutic benefit in spinal and bulbar muscular atrophy [227,228,229]. In addition, chronic treatment with a proprietary Hsp90 inhibitor compound OS47720 offers synaptic protection in symptomatic Tg2576 mice, a model of AD, through a heat shock-like response [230].

#### 5.2.2. Natural Compounds that Induce/Co-Induce Chaperons and Are Applied for Treatment of NDDs

Resveratrol (3,5,4′-trihydroxy-trans-stilbene), a polyphenolic compound of grape skin and seed has antioxidant properties able to scavenge free oxygen, lipid radicals [231], and protects DNA from oxidative damage [232]. In addition, resveratrol has chaperon co-inducer activity as it induces Hsp27, Hsp70, and Hsp90 mRNA expression upon heat stress [233]. Resveratrol decreases the amyloidogenic cleavage of APP, enhances clearance of amyloid beta-peptides and reduces Aβ aggregation [234,235]. It inhibits the hyperphosphorylation of tau protein at Thr181 in a dose-dependent manner via suppression of the activity of glycogen synthase kinase (GSK-3β) and calmodulin-dependent protein kinase II (CaMKII) [236], and at Ser396 through a yet unknown mechanism [237]. In addition, it significantly induces protein phosphatase 2 A (PP2A) activity thereby reducing tau phosphorylation at PP2A-dependent epitopes [238].

Similar to other biophenols, resveratrol inhibits tau fibrillization and/or aggregation along with inhibition of unfolding of tau from microtubule and ceases further tangle formation [239]. In combination with vitamin D, resveratrol successfully prevents cognitive decline in senescence-accelerated mouse-prone 8 (SAMP8) mice [240]. This drug combination significantly reduces soluble Aβ42 level and BACE1 protein expression, phosphorylation of tau at serine404 and p-p53, and in parallel enhances p-CREB protein expression. In addition, this drug combination significantly reduces GFAP and NF-κB p65 levels [240]. A recent 52-week human clinical trial demonstrated that resveratrol modulates neuroinflammation and induces adaptive immunity. In fact, it markedly reduced MMP9 and increased macrophage-derived chemokine (MDC), interleukin (IL)-4, and fibroblast growth factor (FGF)-2 in CSF, whereas in plasma it increased the levels of MMP10 and decreased the levels of IL-12P40, and IL12P70 [241].

The bioavailability of most of the natural drugs is rather poor thus their therapeutic use is very limited. Therefore, recent research is focused on synthesizing new derivatives of natural compounds to enhance their pharmacological activity and extend their therapeutic application. For that, resveratrol was enzymatically transformed to a biologically active drug, piceatannol, using a variant of cytochrome P450_BM3_ [242]. Piceatannol proved to be a better peroxyl radical scavenger than resveratrol [243]. The substitution of hydroxy groups of resveratrol to methoxy groups greatly enhances the lipophilicity of the resulting molecule, pterostilbene (*trans*-3,5-dimethoxy-4-hydroxystilbene), and thus potentiates its therapeutic application [244,245]. Recent studies indicate that pterostilbene exerts a favorable effect in the protection against age-related diseases including AD (reviewed by Nawaz et al. [246]), protects cells from oxidative stress, aging, and dysregulation of autophagy via acting on various signaling pathways and protein homeostasis, and as such contributes to restore cognitive function during aging [247]. In addition, low doses of pterostilbene substantially improve learning and memory functions in SAMP8 mice, a model of accelerated aging that is increasingly being validated as a model of sporadic and age-related AD [248]. Pterostilbene administration is effective in reversing cognitive behavioral deficits, as well as dopamine release while working memory correlates with pterostilbene levels in the hippocampus. [249].

Curcumin is derived from the rhizome of Southeast Asian plant *Curcuma longa.* The dried and crushed rhizome, known as turmeric powder, is used as food colorant. Turmeric powder consists of several components, such as curcumin (the primary and most abundant constituent, responsible for the vibrant yellow color), demethoxycurcumin, bisdemethoxycurcumin, volatile oils (tumerone, atlantone, and zingiberone), sugars, proteins, and resins [250,251]. Interestingly, the prevalence of AD is relatively low in rural north India compared to China or Western countries [252]. Although Indian people regularly consume turmeric in their daily meal, it is still an open question whether there is a direct correlation between these two facts.

Curcumin upregulates Hsp70 and Sirtuin 3 (SIRT3) in hyperglycemic hepatoma cells. SIRT3 regulates mitochondrial antioxidant defense and improves mitochondrial disorders [253]. Curcuminoids isolated from the *Curcuma longa* show antioxidant, anticholinesterase and antidiabetic activities [254], inhibitory effects on Aβ protein, Aβ precursor protein and β-site APP cleaving enzyme 1 [255], dose-dependent inhibition of Aβ aggregation from Aβ1-42, significant decrease of Aβ secretion [256], while blocking the extension of amyloid fibrils and destabilization of preformed Aβ fibrils in vitro [256]. However, recent molecular modelling calculations indicate structural interactions of Aβ peptide with single curcuminoids [257]. Curcumin decreases hyperphosphorylation of tau protein by down-regulating caveolin-1/GSK-3β in N2a/APP695swe cells as well as in APP/PS1 double transgenic mice [258]. It has a strong anti-inflammatory activity, effectively counteracts the p25-mediated glial activation and pro-inflammatory chemokines/cytokines production, reduces the progression of p25-induced tau/amyloid pathology and in turn ameliorates the p25-induced cognitive impairments in p25Tg mice [259]. Recent results showed that curcumin ameliorates the defective insulin signaling pathway by upregulating insulin-like growth factor (IGF)-1R, IRS-2, phosphatidylinositol-3 kinase (PI3K), p-PI3K, Akt and p-Akt protein expression while downregulating IR and IRS-1. By monitoring the brain glucose metabolism in living AD mice using a micro-PET scanning, it was shown that curcumin improves cerebral glucose uptake [260].

Similar to resveratrol, the bioavailability of curcumin is very low. The daily intake of a considerable amount of turmeric—up to 3.6 g/day—is converted into nanomolar concentrations in the plasma [251]. Pharmacokinetic analysis of curcumin in humans showed that the main metabolites present in the peripheral circulation were curcumin glucuronide and curcumin sulfate [261]. Piperine is a component of *Piper nigrum* that inhibits the human enzymes CYP3A4 and P-glycoprotein which are important for the metabolism and transport of xenobiotics. Co-administration of curcumin with piperine [262] or interaction with lipid layers substantially improves its bioavailability [263]. Turmeric containing the three curcuminoids is currently commercialized as the highly standardized lecithin-formulated extract Meriva^®^, of which the absorption was about 29-fold higher than the unformulated curcuminoids extract [264,265,266,267]. Functionalized nanocarriers, composed of curcumin, *N*-acetyl-l-cysteine-polyethylene glycol (100)-monostearate, and nanostructured lipid carrier (Cur-NAPG-NLC) efficiently enhances the bioavailability of curcumin after oral administration [268]. When mixed with milk-derived exosomes in the presence of 10% ethanol: acetonitrile (1:1), curcumin provides a drug load of 18–24%, which can be delivered effectively. Oral administration of exosomal curcumin (ExoCUR) in Sprague-Dawley rats results in 3–5 times higher levels in various organs versus free agent [269]. The poor absorption, low bio-availability, distribution, and targeted delivery to the affected tissue of interest prompted scientists to search for novel nanoparticle-based approaches (reviewed in Gera et al. [270]), such as encapsulation in liposomes [271], in alginate-chitosan-pluronic composite nanoparticles [272], and in solid-lipid microparticles utilizing bovine serum albumin [273].

Astaxanthin (3,3′-dihydroxy-β,β-carotene-4,4′-dione) is a xanthophyll carotenoid belonging to the large class of terpenes. The primary natural source of astaxanthin is the microalgae *Haematococcus pluvialis* [274] and Phaffia yeast *Xanthophyllomyces dendrorhous*, but it is also present in plankton, shrimp, krill, and salmon [275]. Astaxanthin has a very strong antioxidant property due to its unique molecular structure of hydroxyl and keto moieties on each ionone ring [276]. Oxidative stress plays an important role in normal aging and in the pathogenesis of several diseases such as cardiovascular disease, neuroinflammation, and neurodegenerative disease. The primary role of astaxanthin is to eliminate the excess of reactive oxygen species and thereby protects different tissues from oxidative stress [277]. Neuroinflammation starts with microglia activation during which microglia release nitric oxide which interacts with superoxide to form peroxynitrite, a very aggressive reactive oxygen species that will damage proteins, lipids, and DNA [278,279]. Astaxanthin dose-dependently increases Hsp32 and Hsp70 levels during oxygen glucose deprivation—a well-accepted model of neuronal cell death—in SH-SY5Y cells. [280]. Dietary supplementation with astaxanthin and emodin improves the anti-oxidative capabilities, increases hepatic Hsp70 levels, and resistance to acute crowding stress of yellow catfish [281]. Astaxanthin protects PC12 cells from damaging effects of Aβ1-25 at multiple levels, specifically by suppressing the majority of reactive oxygen species, securing the cell viability, inhibiting the expression of Bax caspase 3 and the nuclear translocation of NF-κB, eliminating the elevation of interleukin-1beta and tumor necrosis factor-alpha, suppressing the phosphorylation of p38 mitogen-activated protein kinase, and abolishing the calcium ion influx to effectively maintain calcium homeostasis [282].

Clinical trials on human subjects with mild cognitive impairments demonstrate that astaxanthin in 12 to 20 mg/daily dose substantially improves selective attention, memory functions, and learning [283,284,285]. Recent evidence indicates that astaxanthin promotes neurogenesis and plasticity. Neurogenesis is now widely accepted to occur throughout adulthood, primarily in two regions of the brain: the subventricular zone and the subgranular zone of the dentate gyrus of the hippocampus. Because the hippocampus is essential for learning and memory, neurogenesis likely plays a role in these cognitive processes [277].

Astaxanthin has very poor aqueous solubility resulting in low bioavailability which presents major concerns in product development for oral use [286]. Currently, astaxanthin is available commercially in 10% oleoresin capsules for human use. To date, several methods have been developed for enhancing solubility and bioavailability of bioactives (reviewed Nalawade and Gajjar [286] which includes incorporation in phosphatidylcholine liposomes [287], complexation with calcium ions [288], inclusion complexation with beta-cyclodextrin [289], anti-solvent re-crystallization [290], microencapsulation in a chitosan matrix [291,292] or in rapeseed oil bodies [293], formulation of polysaccharides-based nanoparticles [294], and fenofibrate lipid-based solid dispersion [295]. One of the most promising approaches to enhance dissolution and bioavailability of hydrophobic bioactives is the use of solid dispersion [296]. Solid dispersion is a process during which the hydrophobic drug is dispersed in an inert water-soluble carrier at solid state. Recently, solid dispersions have frequently been prepared by spray-drying method [286,297,298]. Spray drying is a method of producing a dry fine powder from a bioactive-carrier solution by rapidly evaporating solvents with a hot gas. Recent attempts using carotenoid (astaxanthin or lycopene) nanoemulsions obtained by high pressure homogenization resulted a highly bioaccessible pharmacological product [299].

Celastrol is a pentacyclic triterpenoid isolated from the root extracts of *Tripterygium wilfordii* (Thunder god vine) and *Celastrus regelii*. It was demonstrated that celastrol activates HSF1, the master regulator of Hsp gene transcription [300], and induces Hsp70 within dopaminergic neurons [301]. Low dose co-application of celastrol and arimoclomol induces HspA1A (Hsp70-1) and HspA6 (Hsp70B’) as such enhancing the ability of differentiated SH-SY5Y neuronal cells to survive heat shock. Small interfering RNA (siRNA) knockdown of HspA6 and HspA1A results in loss of the protective effect of co-application of celastrol and arimoclomol [300]. Celastrol is a potent inhibitor of lipid peroxidation [302,303] and inhibits induced NO production in mouse brain endothelial cells in a dose-dependent manner [304]. Low concentrations of celastrol (50–100 nM) significantly suppress class II MHC expression induced by LPS [304]. In an earlier experiment of Cleren et al., mice were treated with celastrol before and after injections of MPTP, a dopaminergic neurotoxin, which produced a model of PD. In this model, a 48% loss of dopaminergic neurons induced by MPTP in the substantia nigra pars compacta is significantly attenuated by celastrol treatment. Moreover, celastrol treatment significantly reduces the depletion in dopamine concentration induced by MPTP [301]. Celastrol has been found to improve learning, memory, and psychomotor activity in a rat model of AD [304].

The bioavailability of celastrol is controversial. In adjuvant arthritis, celastrol is bioavailable following oral administration, and efficacy in cognitive tests in rats indicates that the drug can enter the central nervous system [304]. Short-term administration to experimental animals has not revealed serious toxicities, and extracts containing celastrol have been administered for many years to Chinese patients without evidence of carcinogenicity or other limiting side effects [305]. However, according to recent reports, celastrol suffers from many limitations that handicap its clinical utility such as limited aqueous solubility and poor gastrointestinal absorption which resulted into its low oral bioavailability [306]. This led to the development of self-assembled phytosomal nanocarriers (CST-PHY) for improving the solubility and oral bioavailability of celastrol. Pharmacokinetic studies in rabbits revealed significant improvement in CST-PHY oral bioavailability compared with crude celastrol, as evidenced by a 4-fold increase [306].

Chitosan is a polycationic oligosaccharide, the linear polymer of D-glucosamine in b (1–4) linkage. Chitosan treatment results in a dose-dependent induction of Hsp-70 in NT2 neurons [307]. It has an antioxidant and anti-inflammatory activity as it increases the glutathione level through γ-GCS, it dose-dependently enhances the level of Nrf2 protein, and decreases the level of NF-κB in NT2 neurons. In addition, chitosan treatment suppresses oxidative stress-induced Aβ formation in NT2 neurons [307]. Piperine loaded on chitosan-sodium tripolyphosphate nanoparticles (CS-STPP NPs) reduced neuronal loss and astrocyte activation in a chemical kindling model of epilepsy [308].

Teprenone or geranylgeranylacetone (GGA), an acyclic polyisoprenoid widely used for ulcer therapy, induces Hsp70 [309], HspB8, and HspB1 expressions [310]. Via Hsp70 induction, GGA exhibits cytoprotective, anti-inflammatory [311], and antioxidative effects [312]. GGA suppresses *N*-Methyl-*N*-nitrosourea-induced photoreceptor cell loss in mice [313] and confers cytoprotective effects on retinal ganglion cell degeneration using normal tension glaucoma mouse and rat models [314,315]. GGA reduces neuronal death, secondary degeneration, progressive necrosis, and cavitation after spinal cord injury [316]. In addition, GGA induces neuroprotection through the selective mitoK (ATP) channel and the PI3K/Akt pathway [317]. A single oral dose of GGA induces the PI3K/Akt pathway and vascular endothelial growth factor (VEGF) which mediates neuroprotection against kainic acid-induced neuronal cell death [318]. In addition, oral GGA-induced Hsp70 expression induces protein kinase C (PKC) delta, whereas GGA pretreatment enhances ischemia-induced Hsp70, both of which are prevented by pretreatment with chelerythrine, a specific PKC inhibitor. These results suggest that a single oral dose of GGA induces PKC delta and promotes Hsp70 expression in the brain and that GGA plays an important role in neuroprotection against cerebral ischemia [319].

### 5.3. Targeting UPS, and Autophagy Dysfunction in NDDs

Since UPS is a very complex system, chance is high that one of its components is dysregulated what ultimately results in various diseases such as cancer and NDDs. As such, ubiquitination steps and the whole UPS are attractive drug targets. Interestingly, so far only a small number of drugs targeting the UPS have been approved [320]. E1 activating enzymes were targeted and many E1 inhibitors have been developed, but only one (MLN4924) entered clinical trials [321]. E2 conjugating enzymes and E3 ligases have also been targeted [320]. Two proteasome inhibitors, bortezomib (Velcade), a peptide boronate, and cartfilzomib (Kyprolis), a peptide oxyketone, were approved by FDA for cancer therapy but they have not yet been used in the therapy of NDDs. The success of proteasome inhibitors suggests a great potential to develop more drugs targeting UPS and the ubiquitin system.

Impairment of autophagy at one of the different regulatory steps contributes to neurodegenerative processes. Hence, autophagy modulation provides rational therapeutic strategies for such neurodegenerative conditions [322]. However, modulation of autophagy alone will most probably not result in satisfactory clinical effects [323]. This is the result of the very interconnected nature of autophagy. Low levels of autophagy generally promote cell survival owing to the degradation of misfolded proteins (downstream effect) [324]. However, extensive autophagy may kill the targeted cells [325]. It is an upstream effect where autophagy deregulation perturbs global proteostasis and thus contributes to disease progression. As a result, some caution is necessary with respect of autophagy modulating drugs in NDD treatment. Clinical implementation of autophagy modulating anti-degenerative drugs demonstrates that their pro-survival effect should be strengthened by con-joined therapies [323].

Intracellular protein aggregates are autophagy substrates. Hence, autophagy is an attractive therapeutic target for NDDs [326] since enhancing the removal of toxic amyloids limits the toxic load in the cells. Autophagy upregulation decreases the toxic accumulation of different mutant proteins such as mutant huntingtin [327] and mutant α-synuclein [328,329]. Simultaneously with the removal of amyloids, autophagy upregulation reduces neuronal susceptibilities to caspase activation and apoptosis [330]. Candidates for pharmacological induction of autophagy act either on mTOR-dependent or mTOR-independent pathways. The first known drug identified as autophagy activator was rapamycin, which was already in clinical use for other indications [327]. Rapamycin inhibits the kinase activity of mTOR. It was used at first for chronic autophagy induction in mouse models of NDDs (HD and AD) [331]. However, the side effects of rapamycin make it unattractive for use in long-term therapy. Other drugs such as clonidine and rilmenidine are imidazoline receptor agonists and regulate autophagy independently of mTOR. Rilmenidine is a safe, centrally acting, antihypertensive drug. It induces autophagy and has protective effects in a mouse model of HD [332]. A good choice for autophagy induction is the dual treatment with mTOR-dependent rapamycin and mTOR-independent lithium [322]. As such, autophagy can be enhanced to a greater extent by the inhibition of both pathways. Dual treatment allows larger safety window before toxic side-effects.

In a broad review of the therapeutic use of autophagy in NDDs, Nixon summarized the following drugs, drug candidates and signaling pathways [331]:

1. mTORC1 (mammalian target of rapamycin) inhibitors (rapamycin, curcumin, resveratrol, latrepirdine)

2. AMPK activation (lithium, trehalose, rilmenidine, plant alkaloids)

3. Autophagosome formation (Beclin-1 mimetics);

4. Selective autophagy: HDAC (histone deacetylase) modulation, HSC70 overexpression/upregulation, LAMP2A overexpression/upregulation;

5. Lysosomal function:

- Cathepsin activation, lipid clearance, lysosome membrane stabilization: by Hsp70, cholesterol modulation, and calpain inhibitors
- pH acidification: by GSK-3β inhibitors (valproate, lithium)
- Lysosomal exocytosis and exosome release by sphingomyelinase 2, phospholipase D and neuraminidase activation.

Beyond these compounds and pathways, Vidal et al. mention in their review some other drug candidates, e.g., spermidine, L690, 330, carbamazepine, and verapamil, showing also the detrimental consequences of enhanced autophagy levels in animal models [324]. In 2016, a novel potent autophagy-enhancing drug candidate (AUTEN-67, autophagy enhancer 67) was found that significantly increases autophagy flux in cell lines and in vivo models [333]. To maximize the effectiveness of therapeutic approaches and minimize the detrimental effects of autophagy enhancers, future studies should first define and prove the nature of autophagic defects in NDDs. Older drugs (already approved for other indications) and novel drug candidates inducing autophagy are tested for their neuroprotective activity. However, clinical trials are still lacking in this field.

## 6. Conclusions

As postmitotic cells, neurons are unable to dilute the concentration of aggregated polypeptides by cell proliferation. Hence, they are particularly vulnerable against these toxic protein deposits. Several pathways are responsible for balancing brain proteostasis. Hsps are responsible for the maintenance of the normal cellular protein homeostasis, whereas protein degradation pathways (ubiquitination and UPS, ERAD, and different autophagy routes) destroy the unnecessary and dysfunctional components that disturb vital cellular functions. However, during aging, the activity and capacity of these mechanisms are decreasing implying that aging itself is the most important risk factor of NDDs.

At present, there are no disease-modifying drugs approved for the treatment of NDDs. However, novel results on the mechanisms of neurodegeneration and neuroprotection give hope for inventing rational methods for NDD prevention and treatment. According to model animal experiments, chaperon inducers and co-inducers have therapeutic potential in the treatment of AD and related NDDs. Natural compounds such as resveratrol, curcumin, astaxanthin, and celastrol have antioxidant and chaperon co-inducer activity. Unfortunately, their water solubility and bioavailability are low to very low and thus the formulation of these compounds should be solved. Hydroximic acid derivatives such as bimoclomol, arimoclomol, BGP-15, and NG-094 are Hsp co-inducers which act on lipids and may as such stabilize the structure of cellular membranes. As membrane intercalating compounds, the hydroximic acid derivatives belong to a new class of potential drugs for use in “membrane lipid therapy”. For instance, the company Orphazyme is developing arimoclomol in four indications. Phase II/III trials of arimoclomol are running in, among others, ALS and will be completed in 2020.

Activation of the protein degrading systems, UPS, and several autophagy pathways for prevention and treatment of NDDs are currently extensively studied. The success of proteasome inhibitors in cancer therapy gives hope for the successful use of similar compounds in NDD treatment. The dual role of autophagy (cell protecting effect by degrading toxic amyloid proteins and cell damaging by overactivation/deregulation) should be taken into account while designing autophagy-enhancer drugs. Dual treatment of NDDs with mTOR-dependent and mTOR-independent drugs might be a good compromise for reaching larger safety.

The results with small molecule compounds such as Hsp-coinducers (e.g., hydroxymic acid derivatives) and autophagy enhancers are encouraging as they provide evidence for successful targeting the own defense system of the cells for treatment of NDDs. The biggest problem of NDDs is the late diagnosis, since, in an advanced stage, many neurons are dead and beyond rescue. Hence, early diagnosis and the use of multiple drugs may improve the therapeutic success of Hsp-coinducers and UPS and autophagy-modulating drugs.

## Figures and Tables

**Table 1:** Several protein-conformational, neurodegenerative diseases as well as their typical misfolded proteins.

List of Diseases	Misfolded Proteins
Alzheimer’s disease	β-amyloid (Aβ)
	hyperphosphorylated Tau (pTau)
	α-synuclein
Parkinson’s disease	α-synuclein
Huntington’s disease	huntingtin
Lewy-body dementia	α-synuclein
Amyotrophic lateral sclerosis	TDP-43
Prion diseases	superoxide dismutase (SOD)
	prion protein (PrP^sc^)

**Table 2:** Pathways for balancing proteostasis in the neurons.

Name of the Process/Pathway	Localization	Participating Players, Structures
Ubiquitin-proteasome system (UPS)	cytoplasm, proteasome	E1, E2, E3 enzymes, ubiquitin, UBD adaptors, proteasome
ER-associated degradation (ERAD)	ER, cytoplasm, proteasome	Recognition proteins, E3 ligase complex, Doa10 and Hrd1 complexes, ubiquitin, proteasome
Autophagy		
Chaperon-mediated autophagy (CMA)	cytoplasm, lysosome	Cytosolic chaperon, protein-translocation complex LAMP2A, Hsp90AA1, GFAP, lysosomal enzymes
Macroautophagy	cytoplasm, lysosome	Aggresome, phagophor, autophagophor, lysosomal hydrolyses
Microautophagy	cytoplasm, lysosome	HspA8, late endosome, ESCRT for transport, lysosomal hydrolyses

ER, endoplasmic reticulum; UBD, ubiquitin-binding domains; LAMP2A, lysosome-associated membrane protein 2; GFAP, glial acidic fibrillary protein; ESCRT, endosomal sorting complexes required for transport.

## Abbreviations

Aβ	β-amyloid peptide
AD	Alzheimer’s disease
ALS	amyotrophic lateral sclerosis
AMPK	5′-AMP-activated protein kinase
APP	β-amyloid precursor protein
CMA	chaperon mediated autophagy
ER	endoplasmic reticulum
ERAD	endoplasmic reticulum associated degradation
Hsp	heat shock protein
HSF	heat shock factor
HD	Huntington’s disease
LAMPA2A	lysosome-associated membrane protein 2
LSD	lysosomal storage disorder
mTOR	mammalian target of rapamycin
NDD	neurodegenerative disease
PD	Parkinson’s disease
ULK	uncoordinated-51(unc-51) like kinase
UBD	ubiquitin binding domain
UPR	unfolded protein response
UPS	ubiquitin-proteasome system
